# An elevated serum alkaline phosphatase level in hepatic metastases of grade 1 and 2 gastrointestinal neuroendocrine tumors is unusual and of prognostic value

**DOI:** 10.1371/journal.pone.0177971

**Published:** 2017-05-31

**Authors:** Maeva Andriantsoa, Solene Hoibian, Aurelie Autret, Marine Gilabert, Anthony Sarran, Patricia Niccoli, Jean-Luc Raoul

**Affiliations:** 1 Department of Medical Oncology, Paoli-Calmettes Institute, Marseille, France; 2 Department of Biostatistics, Paoli-Calmettes Institute, Marseille, France; 3 Department of Medical Imaging, Paoli-Calmettes Institute, Marseille, France; 4 Aix-Marseille University, Marseille, France; Institut national de la recherche scientifique, CANADA

## Abstract

**Background:**

In our clinical practice we have observed that despite a high hepatic metastatic tumor burden, serum alkaline phosphatase (AP) levels are frequently normal in cases of metastatic neuroendocrine tumor (NET).

**Patients and methods:**

We retrospectively reviewed the records of patients with grade 1 and 2 NETs with liver metastases but without bone metastases seen at our institution in 2013. In total, 49 patients were included (22 female), with a median age of 60 years (range: 28 to 84 years). The primary tumors were located in the duodenum/pancreas (n = 29), small bowel (n = 17) or colon/rectum (n = 3); 10 cases were grade 1 and 39 grade 2. Hepatic involvement was bulky, with more than 10 lesions in 23 patients and a tumor burden above 10% of the liver volume in 26 patients.

**Results:**

Serum AP levels were elevated (≥ upper limit of normal (ULN)) in 16 patients. In multiparametric analysis, elevated serum AP levels were not associated with the primary site, grade, or number or volume of metastases. In multiparametric analysis, progression-free survival was only correlated with grade (p = 0.010) and AP level (p = 0.017).

**Conclusions:**

Serum AP levels are frequently normal in liver metastases from NET, even in the event of a major tumor burden, and the serum AP level can be of prognostic value.

## Introduction

NETs emerging from neuroendocrine cells of the gastrointestinal tract and pancreatic islets are not rare but are frequently found when patients develop metastases[1]. Their clinical course is highly variable[2], and in most cases, the disease is indolent and slowly progressive. Many prognostic factors have been described to better identify patients who will require immediate aggressive treatment versus those who can be treated with long-acting somatostatin analogs[3] or close follow-up. The major prognostic factors are differentiation and the tumor grade defined by the Ki67 index according to Rindi et al classification[4]. Although all grade 3 tumors (Ki67 > 20%) are treated quickly with systemic chemotherapy, the treatment approach is not as clear for grade 1 and 2 tumors. Many other prognostic factors have been proposed for grade 1–2 patients, including the number of liver metastases, tumor slope, primary resection[5], chromogranin A level[6], imaging technique, such as Fluorine-18-fluorodesoxyglucose Positron Emission Tomography (F18-FDG-PET)[7,8] or 68Ga-DOTATOC Positron Emission Tomography/Computed Tomography (68Ga-DOTATOC-PET/CT)[9,10]. In the case of cholestasis, serum alkaline phosphatase (AP) levels are usually elevated either due to alteration of liver function or to extrahepatic dilatation of bile ducts (e.g., tumor, gallstone) but also due to intrahepatic growth of malignant tumors (primary or secondary). In our clinical practice, we have frequently observed patients with liver metastases from gastrointestinal NETs that had normal serum AP levels despite a high hepatic tumor burden. We also observed that normal AP values at diagnosis are often related to indolent, well-differentiated, slowly progressing NETs. The aim of this retrospective study was to compare AP values and tumor burden and to analyze the prognostic value of the AP level in gastrointestinal grade 1 and 2, well-differentiated NET with liver metastases.

## Materials and methods

In this retrospective study, we collected data from all patients seen at our institution between January and December of 2013, some for initial diagnosis and others for follow-up of a grade 1 or 2, well-differentiated metastatic NET from a digestive primary tumor with predominant liver metastases. The inclusion criteria were as follows: age greater than 18 years, histological diagnosis of well-differentiated NET and grade 1 or 2 (Ki67 < 20%) after pathologic review of all cases in the RENATEN-TENPath network (French clinical and pathological networks dedicated to NET). Exclusion criteria were bone metastasis, previous or current anticancer treatment (except somatostatin analogs) and previous hepatic (chemo) embolization. At the date of diagnosis of liver metastases, we collected information about the patients’ characteristics (age, sex), tumor parameters (location of the primary tumor, Ki67, and grade), serum AP level and outcome (date of progression and survival). All CT scans were reviewed to semi-quantitatively assess the tumor burden (metastases representing <10%, 10–50% or >50% of the total liver volume) and determine the number of liver metastases (≤ 10 and >10). Radiological follow-up was only based on CT scans every 3 to 6 months and on RECIST 1.1 for evaluation.

Demographic, patient and tumor characteristics at diagnosis were summarized using median and extreme values for quantitative data and counts and percentages for qualitative data. Differences between groups were tested using the Wilcoxon test for continuous variables and the Chi-squared test for categorical variables. The primary objective of this retrospective study was to assess the prognostic value of an elevated serum AP level for disease progression. Initially, logistic regression was applied to test correlations between AP values and other factors with the Wald test when the AP level was considered a categorical variable; linear regression and the Student t-test were used when the AP level was considered a continuous variable. Overall survival (OS) and progression-free survival (PFS) were calculated from the date of diagnosis of liver metastases and the assessment of the serum AP level to the date of death for OS and to disease progression or death from any cause for PFS. Patients free of an event at the end of follow-up were considered censored at the date of the last follow-up.

Prior to data analysis, the serum AP (> 1ULN vs ≤ 1ULN) level and major prognostic factors were categorized using predefined cut-offs: the number of liver metastases (≤10 vs >10), tumor volume (< 10% vs [10%, 50%] vs > 50%), location of the primary tumor (duodenum/pancreas vs other locations) and grade (G2 vs G1). Probabilities for PFS were calculated using the Kaplan-Meier method, and univariate associations between the factors mentioned above were evaluated with the log-rank test. Multivariate Cox proportional hazards regression was used to evaluate the significance of potential prognostic factors.

All statistical analyses were performed using SAS software (Release 9.3, SAS Institute, Inc, Cary, NC). The level of statistical significance was set to 0.05, with no adjustment for multiplicity.

The study was approved by the Paoli-Calmettes Institutional Review Board and registered as PAlcTNE-IPC 2015–024. As this study was retrospective using previously recorded data, no signed consent from patients was required.

## Results

In the 12-month period, 49 patients meeting the inclusion/exclusion criteria were included in the analysis. In addition to patients who received loco-regional or systemic treatment, the main reasons for exclusion were the presence of bone metastasis (n = 14), which were frequently observed because bone magnetic resonance imaging (MRI) is systematically performed at diagnosis in our institution, and incomplete data (no initial AP level measurement) in 9 patients.

The 22 female and 27 male patients (Table 1) had a median age of 60 years (range: 28–64). The tumors were grade 1 in 10 cases and grade 2 in 39 cases (80%); the median Ki67 value was 5% (range: 1–18). The primary tumors were in the duodenum/pancreas in 29 patients (59%; 2 duodenal tumors grade 2; 27 patients with pancreatic tumors, 4 grade 1 and 23 grade 2), in the small bowel in 17 patients (5 patients with grade 1 and 12 with grade 2), and in the colon/rectum in 3 patients (1 patient with grade 1 and 2 with grade 2) (p = 0.379). There were below or equal to 10 liver metastases in 26 patients (53%, 5 patients with grade 1 and 21 with grade 2) and more than 10 in 23 patients (47%, 5 patients with grade 1 and 18 with grade 2) (p = 0.828). The tumor burden was estimated as < 10% of the total liver volume in 23 patients (47%, 4 patients with grade 1 and 19 with grade 2), between 10 and 50% in 18 patients (37%, 6 patients with grade 1 and 12 with grade 2) and over 50% in 8 patients (16%, all with grade 2) (p = 0.133).

**Table 1:** Characteristics of 49 patients with liver metastases from gastrointestinal neuroendocrine tumor grade 1 and 2; AP: Alkaline phosphatase, CGA: Chromogranin A, 5HIAA: Urinary 5 hydroxy-indole acetate acid; ULN: Upper limit of normal; mets: Metastases; D-Pas: Duodeno-pancreas. P values are given according to the Wilcoxon test for continuous variables and the Chi-squared test for categorical variables.

			All	Grade 1	Grade 2	p	Bowel	D-Pas	p
			N = 49	N = 10	N = 39		N = 20	N = 29	
**Sex**	Male	n (%)	27 (55)	5 (50)	22 (56)	0.72	9 (45)	18 (62)	0.24
	Female	n (%)	22 (45)	5 (50)	17 (44)		11 (55)	11 (38)	
**Age**		Mean (SD)	59.9 (10)	56.8 (12)	60.7 (10)	0.36	59.6 (8)	60.2 (12)	0.64
		Median	60	52.5	60		59.5	60	
		(min-max)	(28–84)	(35–72)	(28–84)		(48–72)	(28–84)	
**AP**		Mean (SD)	1.34 (1.5)	1.0 (0.6)	1.4 (1.7)	0.78	1.1 (0.6)	1.5 (1.9)	0.27
**(XULN)**		Median	0.80	0.80	0.80		0.90	0.80	
		(min-max)	(0.3–9.0)	(0.3–2.2)	(0.3–9.0)		(0.6–2.3)	(0.3–9.0)	
	≤ 1	n (%)	33 (67.3)	7 (70)	26 (66.7)	0.84	12 (60)	21 (72)	0.36
	>1	n (%)	16 (32.6)	3 (30)	13 (33.3)		8 (40)	8 (27.6)	
**CGA**		n	29	9	20	0.09	11	18	0.09
**(XULN)**		Mean (SD)	10.1 (19)	7.7 (19.6)	11.2 (19.9)		7.7 (17.6)	11.5 (21)	
		Median	2.0	0.9	5.0		0.9	4.25	
		(min-max)	(0.3–88)	(0.3–60)	(0.3–88)		(0.3–60)	(0.8–88)	
**5HIAA**		n	24	6	18	0.6	13	11	0.01
**(XULN)**		Mean (SD)	2.5 (3.0)	2.9 (3.6)	2.3 (2.9)		3.8 (3.5)	0.8 (0.5)	
		Median	1.1	1.4	1.1		3.0	0.5	
		(min-max)	(0.2–10)	(0.5–10)	(0.2–10)		(0.2–10)	(0.5–2)	
**Number**	≤ 10	n (%)	26 (53)	5 (50)	21 (54)	0.83	9 (45)	17 (59)	0.35
**of mets**	>10	n (%)	23 (47)	5 (50)	18 (46)		11 (55)	12 (41)	
**Tumor**	< 10%	n (%)	23 (47)	4 (40)	19 (49)	0.13	7 (35)	16 (55)	0.26
**burden**	10–50%	n (%)	18 (37)	6 (60)	12 (31)		10 (50)	8 (28)	
	>50%	n (%)	8 (16)		8 (20.5)		3 (15)	5 (17)	
**Ki 67 (%)**		Mean (SD)	7.4 (5.0)	1.5 (0.5)	8.9 (4.6)		6.4 (4.8)	8.0 (5.2)	0.21
		Median (min-max)	5 (1–18)	1.5 (1–2)	10 (3–18)		5 (1–15)	6 (1–18)	
	< 10%	n (%)	37 (75)	10 (100)	27 (69)	0.043	16 (80)	21 (72)	0.54
	>10%	n (%)	12 (24.5)		12 (30.8)		4 (20)	8 (27.6)	
**Grade**	1	n (%)	10 (20.4)				6 (30)	4 (13.8)	0.17
	2	n (%)	39 (79.6)				14 (70)	25 (86)	
**Primary**	Bowel	n (%)	20 (40.8)	6 (60)	14 (35.9)	0.17			
	D-Pas	n (%)	29 (59.2)	4 (40)	25 (64.1)				

**Fig 1:**
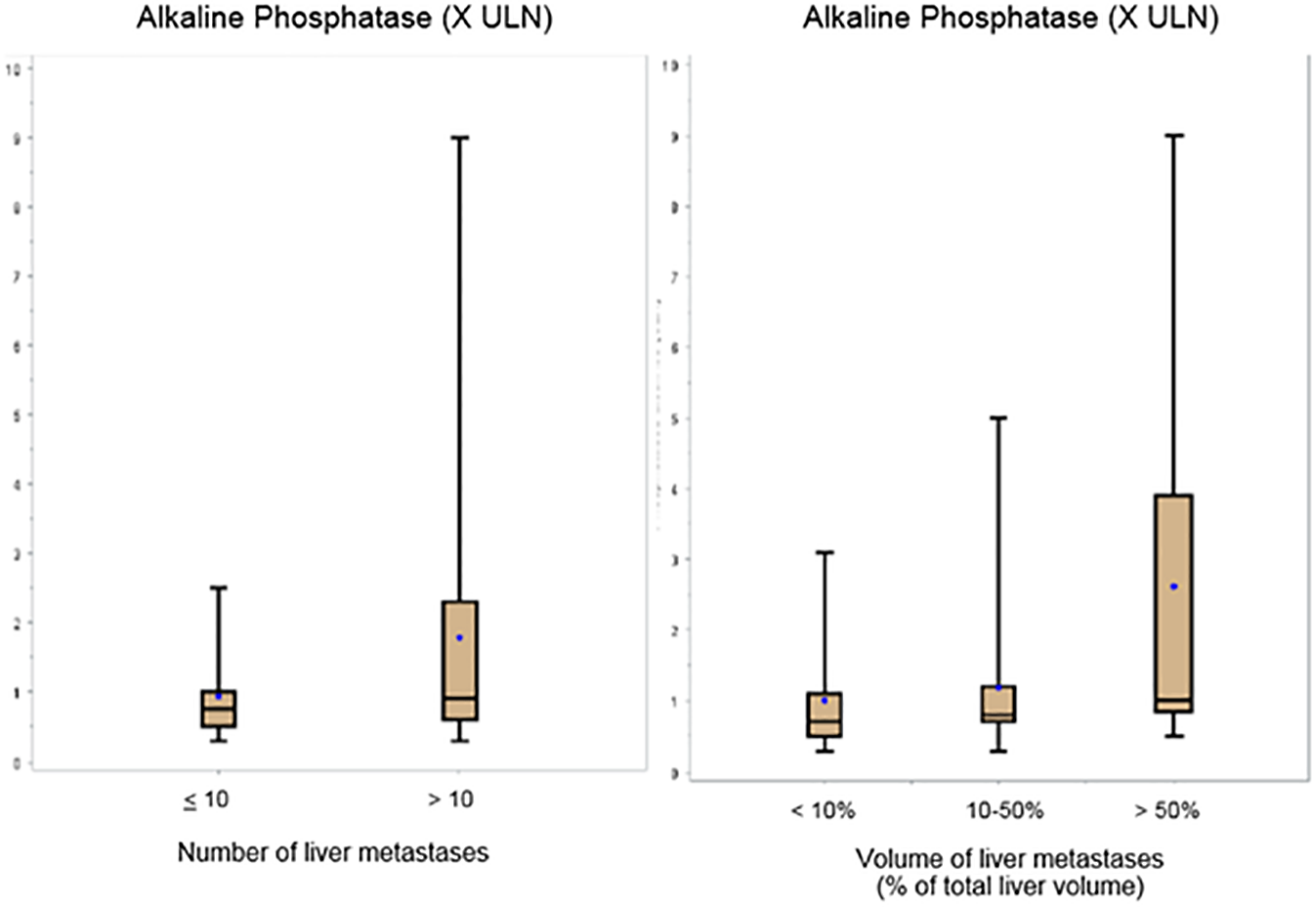
Comparison of AP values (in X ULN) follows the number of liver metastases (left panel) or tumor burden as a percentage of the total liver volume (right panel). Bar: median value, dot: mean value.

The initial serum AP level was normal in 33 patients (67%, 7 with grade 1 and 26 with grade 2), and only 16 patients (33%, 3 patients with grade 1 tumor and 13 with grade 2) had a serum level higher than ULN (p = 0.841). The median AP value was 0.8 ULN (range: 0.3–9.0). When the AP level was considered as categorical variable (normal vs superior to ULN), there was no correlation between the AP value and the following variables: tumor site (p = 0.365), tumor grade (p = 0.841), number of metastases (p = 0.133) and tumor volume (p = 0.473). The median AP values (X ULN) were 0.70 (range: 0.3–3.1), 0.80 (range: 0.3–5.0) and 1.0 (range: 0.5–9.0) in patients with a liver tumor volume less than 10% of the total liver volume, between 10 and 50% and higher than 50%, respectively; and the median AP values were 0.75 (range: 0.3–2.5) and 0.90 (range: 0.3–9.00) for cases with below or equal to 10 and more than 10 liver metastases, respectively (Fig 1). When the AP level was considered a continuous variable, only the tumor burden was correlated with the AP value (p = 0.028).

**Fig 2:**
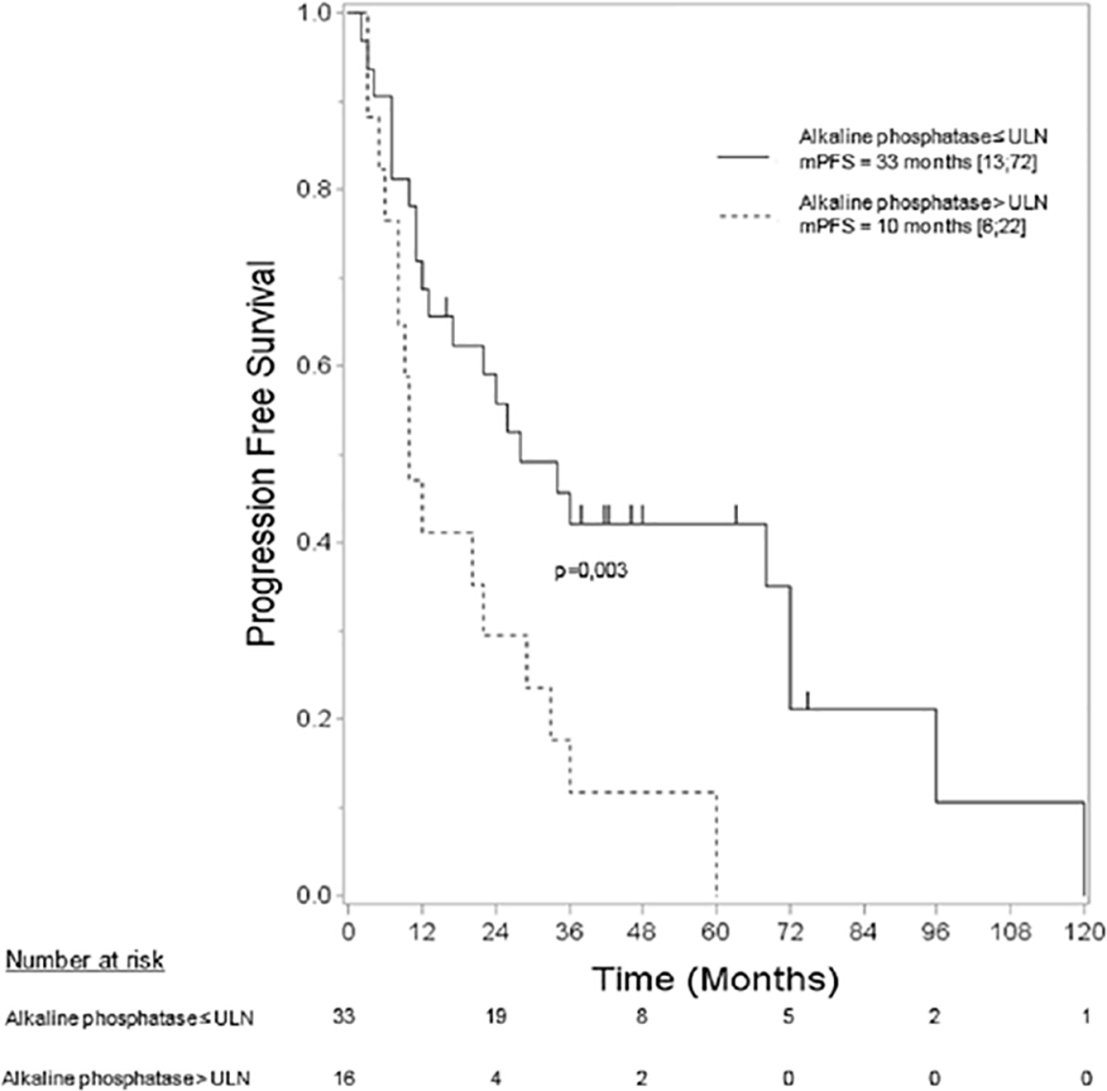
Progression-free survival follows the alkaline phosphatase value.

At the end of the follow-up period, 40 patients had progressed (5 patients with grade 1 tumor and 35 with grade 2), and 18 had died (1 with grade 1 and 17 with grade 2). The median OS was not reached, and the 5-year OS was 59% (CI95%: [42; 73]). The median of PFS was 22 months (CI95%: [11; 34]), and the 5-year PFS was 24% (CI95%: [12; 38]). By univariate analysis (Table 2), PFS was related to the number of liver metastases (p = 0.039), grade (p = 0.005) and AP value (p = 0.003). The tumor burden was not significant but appeared to show a trend (≤10% vs >10%, p = 0.066), and the location of the primary tumor was not significant (p = 0.698). The median PFS was 10.0 months (CI95%: [6; 22]) when the AP was above the ULN and 33.0 months (CI95%: [13;72]) when the AP was normal (Fig 2). According to multivariate analysis of PFS with the AP value, the number of liver metastases, tumor volume, location of the primary tumor and grade as covariates, only the grade (p = 0.010) and AP value (p = 0.017) were independently associated with PFS. None of the above mentioned factors was correlated with OS.

**Table 2:** Univariate and multivariate analyses of prognostic factors for progression-free survival. Univariate and multivariate Cox regressions, with Kaplan-Meier estimations of the median, hazard ratio and p-value according to log-rank tests for progression-free survival. Nbr: number; Obs: observations; ULN: upper limit of normal.

					*Univariate analysis*		*Multivariate analysis*	
	*Classes*	*Nbr of obs*	*Nbr of events (%)*	*Median in months [CI 95%]*	*P-value Log-rank*	*Hazard Ratio [CI 95%]*	*P-value Log-rank*	*Hazard Ratio [CI 95%]*
**AP level**	≤ULN	33	24 (73%)	33 [13.0, 72.0]	0.003	1	0.017	1
	>ULN	16	16 (100%)	10 [6.0, 22.0]		2.63 [1.34,5.14]		2.49 [1.18,5.25]
**Location of the primary**	other locations	20	16 (80%)	28 [10, 60]	0.698	1	0.268	1
	Duodenum/pancreas	29	24 (83%)	12 [7.0,33.0]		1.13 [0.59,2.17]		1.50 [0.73,3.09]
**Number of liver metastases**	≤ 10	26	20 (77%)	33 [22, 68]	0.039	1	0.720	1
	> 10	23	20 (87%)	10 [7, 20]		1.92 [1.01,3.64]		1.20 [0.44,3.26]
**Tumor volume**	< 10	23	16 (70%)	36 [12, 72]	0.066	1	0.202	1
	≥10	26	24 (92%)	14 [8, 26]		1.79 [0.94,3.40]		1.90 [0.71,5.10]
**Grade**	G1	10	5 (50%)	120 [3, 120]	0.005	1	0.010	1
	G2	39	35 (90%)	13 [10, 26]		3.93 [1.38,11.15]		4.16[1.40,12.40]

## Discussion

In this small series of liver metastases from gastrointestinal NETs, we confirmed our initial clinical hypothesis, demonstrating that the AP level was elevated only in a minority of cases. Although this increase was not clearly related to the tumor burden, it did exhibit a prognostic value. There are many limitations in this study. It is a retrospective analysis that only focused on patients who did not require loco-regional or systemic treatment and who by definition had slowly progressive tumors. Additionally, the sample size was small. However, our goal was to draw attention to the findings to inspire future prospective prognostic analyses.

Only a few papers to date have evaluated the serum AP level in liver metastases from gastrointestinal NET. The results agree with the low frequency of cases with an elevated serum AP level and with its possible prognostic value. In a series of 137 metastatic NETs[11], including 115 with liver metastases, the AP level was normal in 60% of the cases. In another series of patients planning to receive transarterial chemoembolization for metastatic NETs, only 22 of 109 (including 7 with bone metastases) had an elevated AP level[12]. These data are in accordance with our findings showing that 67% of patients with liver metastases had normal AP values.

To the best of our knowledge, only one other study[11] has analyzed the AP value within the context of the extension of hepatic involvement; the authors also observed that, even when hepatic involvement was above 50%, the mean AP value can remain normal. We failed to demonstrate any relationship between the AP level and Ki67 level or grade. A recent paper focusing on G2 NET(13), including 48% of cases with liver metastases, performed a subgroup analysis based on Ki67 (cut-off of 10% between subgroups); the only difference between these 2 subgroups were AP and chromogranin A levels.

In contrast, all papers evaluating AP levels agree that the AP level is of prognostic value. Clancy et al[11] reported that the AP value was associated with OS. In a series of 109 patients, Onesti et al[12] performed univariate analysis of the prognostic factors for OS and found that the AP level, grade and tumor burden were associated with the prognosis. In multivariate analysis, only the AP level had prognostic value. In a series of 40 grade 2 NET patients by Hauck et al[13], those with a Ki67 value between 3% and 9% had a lower AP level and a better prognosis than those with a higher Ki67 value (10–20%).

Thus, the AP value in liver metastases from NET can reflect the aggressiveness of the disease (speed of metastatic growth) more than the burden and can explain why there is likely a relationship between the serum AP level and outcome.

In conclusion, in this retrospective analysis of patients with liver metastases from grade 1–2 NETs and without bone metastases, the AP level was only elevated in one-third of the patients, and it was not related with the tumor burden (number and volume of metastases). In contrast, by multivariate analysis, the AP level and grade were associated with PFS. These findings must be confirmed in a large prospective series, and the AP level must be considered as part of the initial work-up for metastatic NET.
